# Smooth super-twisting sliding mode control for the class of underactuated systems

**DOI:** 10.1371/journal.pone.0203667

**Published:** 2018-10-03

**Authors:** Sami ud Din, Fazal ur Rehman, Qudrat Khan

**Affiliations:** 1 Department of Electrical Engineering, Capital University of Science and Technology (C.U.S.T), Islamabad, Pakistan; 2 Center for Advance Studies in Telecommunications (CAST), COMSATS University, Islamabad, Pakistan; Lanzhou University of Technology, CHINA

## Abstract

In this article, Smooth Super-twisting Sliding Mode Control (SSTWSMC) is investigated for the class of underactuated system. In underactuated systems, the control design is not directly applicable as for other systems (known as fully actuated systems). Therefore, at initial step, a nonlinear uncertain model of systems is transformed into the controllable canonical form, and then Smooth Super Twisting (SSTW) based Sliding Mode Control (SMC) is devised for the control design purpose for the considered class. In addition, closed loop stability of the proposed technique is presented in a fascinating way. The effectiveness and supremacy of the proposed control technique is proven by extensive analysis between conventional Sliding Mode Control (SMC), Super twisting (STW) sliding mode control and Smooth Super-twisting Sliding Mode Control (SSTWSMC). The comprehensive analysis evaluates the attributes like robustness enhancement, settling time, control effort, chattering reduction, overshoot, sliding mode convergence, etc. and is supported by simulations as well as practical implementation on ball and beam balancer (which is considered as application example).

## Introduction

As by definition, underactuated systems have less number of control input than their degrees of freedom (DOF). Therefore, the control design for such systems are more challenging than other systems (called fully actuated systems). The underactuated systems remain under the spotlight in the control community from the last two decades [1]. Under-actuation can be introduced deliberately for acquiring certain gains like weight reduction in (aerial and underwater vehicles) [2], and it may appear in the dynamics incidentally, like in case of any malfunction [3]. The control design of the aforesaid class has its vital importance in the area of locomotive systems, robotics and different kinds of manipulators, etc. [1–3]. Systems like, ball and beam balancer [4], flexible joint manipulator of 1-link, single and double inverted pendulum also belongs to this class [5]. For the independent or standalone effective operation of such systems, very sophisticated control techniques are required.

From the perspective of control design, a wide range of control strategies are available for fully actuated systems, which is not the case with underactuated systems due to the dissatisfaction with Brockett necessary condition [6]. In addition to this, linearization becomes difficult via smooth feedback [1] for a large class of underactuated systems and control design varies from system to system (in accordance with their dynamics) [7]. In the context of control design, well-established works on the considered underactuated system includes, passivity-based control [8], adaptive control [9], optimal control [10] and feedback linearization [11], etc. However, these control techniques become not fully applicable to a broad class of underactuated systems due to difficulty in linearization [2]. In addition to this, such techniques are not applicable towards flat underactuated systems, e.g., vertical take-off and landing (VTOL) aircraft, inertia-wheel pendulum (IWP) [1]. To overcome this limitation, passivity-based approach is suggested by the researchers of control community [1], but it can only be workable effectively for less than two DOF [7]. Back-stepping technique is proposed to overcome the constraint of passivity-based approach. Due to its reverse substitution design, its implementation towards real (practical) systems is considered to be quite unrealistic [1]. In addition to this, aforementioned strategies are not able to counter the matched disturbances and also redundant in robustness.

In practice, robustness and precision are ever demanding regarding the employment of any control strategy. In this regard, Sliding Mode Control (SMC) based techniques gain considerable attention from control research community (see for instance [1–5,7]). As SMC suffers from high-frequency oscillatory phenomenon (known as chattering), therefore its applicability to real systems is somehow limited. To counter this chattering problem Higher Order Sliding Mode Control (HOSMC) based strategies are also posed by the researchers [2], like Integral Sliding Mode Control (ISMC) [2], Twisting and Super-twisting (STW) algorithms [3] which show appealing results. Global sliding mode control (GSMC) [12–14] and Fast terminal sliding mode technique is also posed for such class, which also shows promising results due to finite time convergence [5].

In this work, smooth super-twisting based sliding mode control is presented for the considered class of underactuated systems. The beauty of Smooth Super-Twisting Sliding Mode Control (SSTWSMC) is the conservation of the features of SMC, while reducing the chattering effect. This SSTWSMC framework guarantees effectiveness in many sensitive applications and provides nearly chatter-free smooth control action. Before the design presentation, initially, a given nonlinear uncertain model of the underactuated system is transformed into an input-output form according to procedure laying in [2], the driving applied control input of the transformed system is then designed via Smooth Super-Twisting (SSTW) based Sliding Mode Control (SMC) strategy. At the second stage, the proposed smooth super-twisting sliding mode control is practically implemented on the ball and beam balancer (considered as an illustrative example for the considered class). In the third phase, extensive analysis of the simulation as well as experimental results with respect to its tracking performance is then conducted considering the parameters like, robustness enhancement, settling time, control effort, chattering reduction, etc.

It is worthy to mention that our contribution in this work is in three folds, i.e., First, the transformation of the system into canonical form by defining a suitable output. Second, is the simulation, as well as practical implementation of smooth super-twisting sliding mode control technique. The comparative analysis of the SSTWSMC with STWSMC and conventional SMC is considered as the third contribution of this work. This paper is organized in the following manner.

General presentation of the considered class and problem statement are presented in Section II. The control law design of the SSTWSMC is presented in Section III. In Section IV, an illustrative example is considered, and the proposed technique is employed in the application example. Simulation and implementation results are also displayed in Section IV. At the end conclusion with comparative results are given, supported by the references.

## Problem formulation

The dynamical equation governs the motion for the class of underactuated systems is represented as follows [5]

$$M(q)\ddot{q}+C(q,\dot{q})\dot{q}+G(q)+F(\dot{q})=B(\rho +\delta (q,\dot{q},t))$$ (1)

where *q*, $\dot{q}$ represent position and velocity, respectively, and both belong to *R*^*n*^. Similarly, inertia, gravitational, fractional and coriolis torque matrices are expressed by *M*(*q*) ∈ *R*^*n*×*n*^, *G*(*q*) ∈ *R*^*n*×*1*^, $F(\dot{q})\in R^{n\times n}$ and $C(q,\dot{q})\in R^{n\times n}$, respectively. Control input channel is displayed as *B*, where *ρ* ∈ *R*^*m*^ (*m* < *n*) is representing the control input, and $\delta (q,\dot{q},t)$ poses the matched disturbance/uncertainty (which entered in to the system via input channel). It is worthy to mention, that the origin is considered to be equilibrium for the aforementioned system and it is assumed that (*M*^−1^(*q*)*B*) is full rank.

Now by pursuing the procedure reported in [15–16], the system in (1), can be rewritten as following transformed form;

$$\dot{x_{1}}=x_{2}$$

$$\dot{x_{2}}=f_{1}(x_{1}{,x}_{2},x_{3},x_{4})$$ (2)

$$\dot{x_{3}}=x_{4}$$

$$\dot{x_{4}}=f_{2}(x_{1}{,x}_{2},x_{3},x_{4})+b(x_{1}{,x}_{2},x_{3},x_{4})\rho$$

In Eq (2), measurable states of the system are represented by *x*_1_,*x*_2_,*x*_3_,*x*_4_, where nonlinear smooth function are represented by *f*_1_ and *f*_2_. It can be clearly observed in (2), the last two equations can be directly controllable, but the first two equations can only be controlled via *x*_2_ and *x*_1_ indirectly.

### Remark 1

As we transformed (1) into a controllable canonical form, it may be employed to other systems like inverted pendulum [5], TORA [16], etc.

By pursuing the procedure laying in [2], system presented in (2) can be transformed as (3)

$$\dot{\xi_{1}}=\xi_{2}$$

$$\dot{\xi_{2}}=\xi_{3}$$

⋮

$$\dot{\xi_{n}}=\varphi (\xi )+\gamma (\xi )\{\rho +\Delta G_{m}(\xi ,t)\}$$ (3)

In the above Eq (3), states vectors are represented by *ξ* = [*ξ*_1_,*ξ*_2_,…,*ξ*_*n*_]^*T*^. Matched disturbances are displayed by Δ*G*_*m*_(*ξ*,*t*), (which is considered to bounded |Δ*G*_*m*_ (*ξ*,*t*)| ≤ *Γ*). *ρ* is the control input applied to the system in (2). Now, our clear aim is to design a controller, which is able to control the system presented by (3), which implies clear solution to the control problem of system (2) lead us to (1). At this stage, the system presented in (3) is considered under regulation problem (i.e. to steer all states to zero). SSTWSMC is employed to perform the desired task. Now, we are ready to purse the control design.

## Control law design

In this section, the control law is designed for system (3). As chattering effect is considered to be major drawback in the conventional sliding mode control technique. Effort has been put to remove/minimize this chattering phenomenon via use of Higher Order Sliding Mode Control (HOSMC) [17]. In this approach, the sliding mode occurs along the intersection of the sliding variable and its derivative of order *r*. By pursuing the procedure of [7] and [17], the sliding set is defined to be $=\dot{\sigma }=\ddot{\sigma }=\dddot{\sigma }=\cdots =\sigma^{\left(r-1\right)}=0$. The beauty of this technique is confirming the enforcement of sliding mode in finite time along the defined sliding set in the presence of the disturbances/uncertainties which in turn results in increase in accuracy of the sliding modes. Moving to one step further, now we aim to devise the smooth super-twisting based sliding model control (collectively abbreviated as SSTWSMC) for the aforesaid nonlinear system.

### Smooth super-twisting sliding mode control (SSTWSMC)

In this design, the sliding set consists of the intersection of hyperplanes *σ*(*ξ*) = 0 and $\dot{\sigma }(\xi )=0$ i.e., the sliding mode occurs on the following set

$$\sigma (\xi )=\dot{\sigma }(\xi )=0$$ (4)

Consider the sliding surface.

$$\sigma (\xi )=\sum_{i=1}^{n}c_{i}\xi_{i}$$ (5)

Now, by taking the time derivative of (5), one gets

$$\dot{\sigma }(\xi )=\sum_{i=1}^{n-1}c_{i}\xi_{i+1}$$ (6)

where *c*_*i*_ > 0 are chosen in such a way that *σ* becomes Hurwitz polynomial. From [18], the respective control law can be referred as:

$$\rho =-k_{1}{\left|\sigma \right|}^{\frac{\mu -1}{\mu }}sign(\sigma )+z$$

$$\dot{z}=-k_{2}{\left|\sigma \right|}^{\frac{\mu -2}{\mu }}sign(\sigma )$$ (7)

Collectively, it can be represented as:

$$\rho =-k_{1}{\left|\sigma \right|}^{\frac{\mu -1}{\mu }}sign(\sigma )-\int k_{2}{\left|\sigma \right|}^{\frac{\mu -2}{\mu }}sign(\sigma )$$ (8)

By choosing

$$\rho =\frac{1}{\gamma (\xi )}[\rho_{eq}+\rho_{s}]$$ (9)

In above equation

$$\rho_{eq}=-c_{1}\xi_{2}-\cdots -c_{i}\xi_{n+1}$$ (10)

$$\rho_{s}=-k_{1}{\left|\sigma \right|}^{\frac{\mu -1}{\mu }}sign(\sigma )+z$$ (11)

where,

$$\dot{z}=-k_{2}{\left|\sigma \right|}^{\frac{\mu -2}{\mu }}sign(\sigma ),\;k_{1},k_{2}>0,\mu \geq 2$$ (12)

By substituting Eq (9) to (12) into (6), ones obtain

$$\dot{\sigma }(\xi )=-k_{1}{\left|\sigma \right|}^{y}sign(\sigma )+z$$

$$\dot{z}=-k_{2}{\left|\sigma \right|}^{y-\frac{1}{\mu }}sign(\sigma ),\;k_{1},k_{2}>0,y=\frac{\mu -1}{\mu }$$ (13)

Define

$$\varsigma^{T}=\lfloor {\left|\sigma \right|}^{y}sign(\sigma )z\rfloor$$ (14)

Then by the following procedure defined in [18], one obtains

$$\dot{\varsigma }=\left[\begin{array}{c} y{\left|\sigma \right|}^{y-1}\dot{\sigma }\\ \dot{z} \end{array}\right]=\left[\begin{array}{c} y{\left|s\right|}^{-\frac{1}{\mu }}\{-k_{1}{\left|\sigma \right|}^{y}sign(\sigma )+z\}\\ -k_{2}{\left|\sigma \right|}^{y-\frac{1}{\mu }}sign(\sigma ) \end{array}\right]$$ (15)

$$={\left|\sigma \right|}^{-\frac{1}{\mu }}\left[\begin{array}{c} y\{-k_{1}{\left|\sigma \right|}^{y}sign(\sigma )+z\}\\ -k_{2}{\left|\sigma \right|}^{y}sign(\sigma ) \end{array}\right]$$ (16)

$$={\left|\sigma \right|}^{-\frac{1}{\mu }}\left[\begin{array}{cc} -yk_{1} & y\\ -k_{2} & 0 \end{array}\right]\left[\begin{array}{c} {\left|\sigma \right|}^{y}sign(\sigma )\\ z \end{array}\right]={\left|\sigma \right|}^{-\frac{1}{\mu }}A\zeta$$ (17)

where,

$$A=\left[\begin{array}{cc} -yk_{1} & y\\ -k_{2} & 0 \end{array}\right]$$ (18)

$$\dot{\varsigma }={\left|\sigma \right|}^{-\frac{1}{\mu }}A\xi .$$ (19)

The eigenvalues of $A=\left[\begin{array}{cc} -yk_{1} & y\\ -k_{2} & 0 \end{array}\right]$ are the roots of Hurwitz polynomial:

$$|\lambda I-A|=\left|\begin{array}{cc} \lambda +yk_{1} & -y\\ k_{2} & \lambda \end{array}\right|=\lambda^{2}+\lambda (yk_{1})+(yk_{2})=0$$ (20)

Therefore $A=\left[\begin{array}{cc} -yk_{1} & y\\ -k_{2} & 0 \end{array}\right]$ is asymptotically stable.

#### Theorem

Consider a Lyapunov function *V* = *ζ*^*T*^*Pζ*, where $P\epsilon R^{2\times 2}$ is the positive definite and symmetric matrix satisfying the Lyapunov equation *A*^*T*^*P* + *PA* = −*Q*, where $Q\epsilon R^{2\times 2}$ is the positive definite and symmetric matrix. Then

$$\dot{V}=-{\left|\sigma \right|}^{-\frac{1}{\mu }}\zeta^{T}Q\zeta <0$$ (21)

Therefore, the system (19) is asymptotically stable. Thus *σ* → 0, therefore *x*_1_,..,*x*_4_ converge to zero. In the upcoming section aforesaid control design strategy is applied to ball and beam balance, taken as an example for the considered class.

## Illustrative example

This section is dedicated to the illustrative example, for the aforesaid class. Ball and beam balancer is considered as an application example for the specified class. In the forthcoming sections, the system description is given along with control design. In addition, simulation and experimental results are also analysed along with an extensive comparative analysis.

### Description of ball and beam balancer

**Fig 1:**
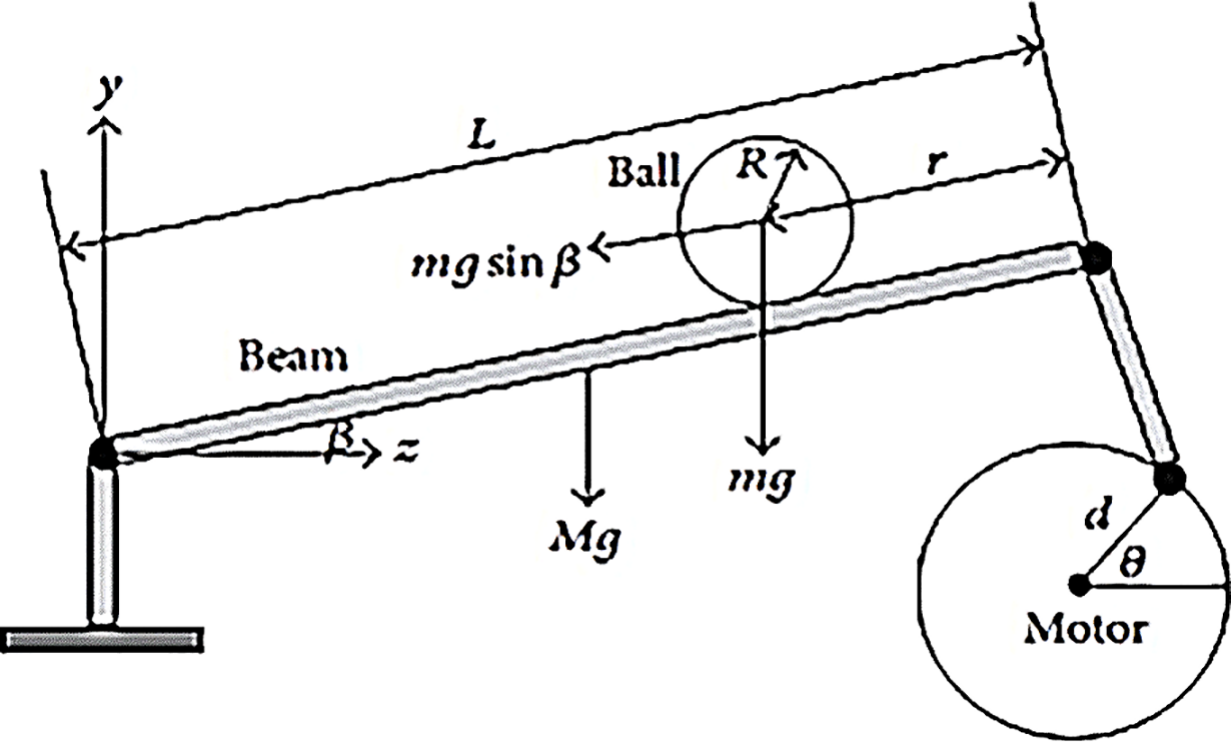
Ball and beam balancer schematic representation.

The ball and beam balancer is very appealing as an example for the considered class, due to its nonlinear nature and a wide range of industrial and military applications. The applications of ball and beam balancer include fuel balancing in rockets and other vertical take-off objects [4], comfort balancing of the passenger cabin in luxuries cars [7]. In addition to such applications, its dynamics allow the flexibility to implement several classical/modern control strategies [2]. It is also considered to be a part of any modern advance control laboratory. (Fig 1) displays the schematic model of ball and beam balancer where its typical parameter are reported in Table 1.

**Table 1:** Parameters and values.

Parameter and description	Values with units
*g-* Gravitational acceleration	9.81 m/s2
*m-* Mass of ball	0.04 kg
*M-* Mass of beam	0.15 kg
*L-* Length of beam	0.4 m
*R*_*m*_*-* Resistance of armature of the motor	9 Ω
*J*_*m*_*-* Moment of inertia of motor	7.35×(10)-4 Nm/(rad/s2)
*C*_*m*_*-* Torque constant of motor	0.0075 Nm/A
*C*_*g*_*-* Gear ratio	4.28
*d-* Radius of the arm connected to a servo motor	0.04 m
*J*_*1*_*-* Moment of inertia of the beam	0.001 kgm2
*C*_*b*_*-* Back *emf* constant value	0.5625 V/(rad/s)

In general, this system is equipped with a beam operated by a motor and ball rolls over the beam freely. This system works on a feedback strategy. Moreover, the beam work as a potentiometer measured the ball position and feedbacked the system. In response the motor acts accordingly to control the position of the ball (for more detail, see [2] and [7]).

The motion governing equations of the ball and beam system are taken from [4] and [7]:

$$(mr^{2}+T_{1})\ddot{\beta }+(2mr\dot{r}+T_{2})\dot{\beta }+(mgr+\frac{L}{2}Mg)\cos\beta =\rho$$

$$T_{4}\ddot{r}-r\dot{\beta^{2}}+gsin\;\beta =0$$ (22)

where *θ*(*t*) is the angle, spans to make the ball stable, the lever angle is represented by *β*(*t*),whereas *r*(*t*) represents the position of the ball on the beam. Motor input voltage is presented by *v*_*in*_(*t*), and mathematically controlled input appears as *ρ*(*t*) = *T*_3_*v*_*in*_(*t*) in the dynamical model. The derived parameters used in Eq (22) are represented by *T*_1_,*T*_2_,*T*_3_, and *T*_4_ with the following mathematical relations [4]

$$T_{1}=\frac{R_{m}\times J_{m}\times L}{C_{m}\times C_{b}\times d}+J_{1}$$

$$T_{2}=\frac{L}{d}\left(\frac{C_{m}\times C_{b}}{R_{m}}+C_{b}+\frac{R_{m}\times J_{m}}{C_{m}\times C_{g}}\right)$$ (23)

$$T_{3}=1+\frac{C_{m}}{R_{m}}$$

$$T_{4}=1.4$$

By assuming *x*_1_ = *r* (position of the ball on a beam), $x_{2}=\dot{r}$, *β* = *x*_3_ (beam angle) and $x_{4}=\dot{\beta }$, one may get

$$\dot{x_{1}}=x_{2}$$

$$\dot{x_{2}}=\frac{1}{T_{4}}(-gsin{(x}_{3}))$$ (24)

$$\dot{x_{3}}=x_{4}$$

$$\dot{x_{4}}=\frac{1}{mx_{1}^{2}+T_{1}}(\rho -(2mx_{1}x_{2}+T_{2})x_{4}-\left(\begin{array}{c} mgx_{1}\\ +\frac{L}{2}Mg \end{array}\right)cos\;x_{3})$$

Now, we are interested in the ball position at beam (*y* = *x*_1_). The presentation of Eq (24) is quite identical to the system shown in (3). In the upcoming section, the control design is outlined.

### Control design

The model presented in (22), can be rewritten as follows by adopting the procedure laying in [2]:

$$y=x_{1}$$ (25)

$$\dot{y}=x_{2}$$ (26)

$$\ddot{y}=-\frac{g}{T_{4}}sin{(x}_{3})$$ (27)

$$y^{\left(3\right)}=-\frac{g}{T_{4}}x_{4}cos{(x}_{3})$$ (28)

$$y^{\left(4\right)}=\frac{1}{T_{4}\left(mx_{1}^{2}+T_{1}\right)}\left[-\rho cos\;x_{3}+\left(2mx_{1}x_{2}+T_{2}\right)x_{4}cos\;x_{3}+\left(mgx_{1}+\frac{L}{2}Mg\right)cos^{2}x_{3}+x_{4}{}^{2}\left(mx_{1}^{2}+T_{1}\right)\sin\;x_{3}\right]$$ (29)

Eq (29) can be rewritten as follows.

$$y^{\left(4\right)}=f_{s}+h_{s}\rho$$ (30)

$$f_{s}=\frac{g}{T_{4}}\left[\begin{array}{c} \frac{(2mx_{1}x_{2}+T_{2})x_{4}+(mgx_{1}+\frac{L}{2}Mg){cos}^{2}x_{3}}{mx_{1}^{2}+T_{1}}\times \cos\;x_{3}+x_{4}^{2}sinx_{3} \end{array}\right],\;h_{s}=\frac{-g}{T_{4}(mx_{1}^{2}+T_{1})}\times \cos\;x_{3}$$

By putting the value of *n* = 4, in the system presenting in (3), one gets

$$\dot{\xi_{1}}=\xi_{2}$$

$$\dot{\xi_{2}}=\xi_{3}$$ (31)

⋮

$$\dot{\xi_{4}}=\varphi (\xi )+\gamma (\xi )\rho$$

where *y*^(*i*−1)^ = *ξ*_*i*_.

$$\varphi (\xi )=f_{s}=\frac{g}{T_{4}}\left[\begin{array}{c} \frac{(2m\xi_{1}\xi_{2}+T_{2})\xi_{4}+(mg\xi_{1}+\frac{L}{2}Mg){cos}^{2}\xi_{3}}{m\xi_{1}^{2}+T_{1}}\\ \times \cos\;\xi_{3}+x_{4}^{2}sin\xi_{3} \end{array}\right]$$ (32)

$$\gamma (\xi )\rho =h_{s}=\frac{-g}{T_{4}(m\xi_{1}^{2}+T_{1})}\times \cos\;\xi_{3}$$ (33)

For *n* = 4 in Eq (5) becomes,

$$\sigma (\xi )=c_{1}(\xi_{1}-r_{d})+c_{2}\xi_{2}+c_{3}\xi_{3}+\xi_{4}$$ (34)

Similarly (6), can be represent as (35), for *n* = 4,

$$\dot{\sigma }(\xi )=c_{1}\xi_{2}+c_{2}\xi_{3}+c_{3}\xi_{4}+\varphi (\xi )+\gamma (\xi )\rho$$ (35)

where *c*_*i*_ > 0 are chosen in such a way that *σ* becomes Hurwitz polynomial. By choosing

$$\rho =\frac{1}{\gamma (\xi )}[\rho_{eq}+\rho_{s}]$$ (36)

In Eq (36)

$$\rho_{eq}=-c_{1}\xi_{2}-c_{2}\xi_{3}-c_{3}\xi_{4}-\varphi (\xi )$$

$$\rho_{s}=-k_{1}{\left|\sigma \right|}^{\frac{\mu -1}{\mu }}sign(\sigma )+z$$ (37)

where,

$$\dot{z}=-k_{2}{\left|\sigma \right|}^{\frac{\mu -2}{\mu }}sign(\sigma ),\;k_{1},k_{2}>0,\mu \geq 2$$

The overall controller becomes

$$\rho =\frac{1}{\gamma (\xi )}[-c_{1}\xi_{2}-c_{2}\xi_{3}-c_{3}\xi_{4}-\varphi (\xi )-k_{1}{\left|\sigma \right|}^{\frac{\mu -1}{\mu }}sign(\sigma )-\int k_{2}{\left|\sigma \right|}^{\frac{\mu -2}{\mu }}sign(\sigma )]$$ (38)

value of *k*_1_,*k*_2_ and *μ* is given in Table 2.

**Table 2:** Parametric values used in the tracking for SSTW.

Constants	C_1_	C_2_	C_3_	K_1_	K_2_	𝜇
**In Simulation**	15	12	9	2.5	4	2.9
**In Experimental framework**	25	9	5	1.5	0.5	3.5

#### Remark 2

It is worthy to mention if *μ* = 2, Smooth Super-twisting Sliding Mode Control (SSTWSMC) reverts to the conventional Super-twisting (STW) sliding mode control.

### Simulation results

The system defined in (22) is operated under the action of the control law (38). For extensive analysis, simulation results of conventional SMC and super-twisting (STW) algorithm reported in [7] are also analysed against Smooth Super-twisting Sliding Mode Control (SSTWSMC). The gains used in SSTWSMC controller during simulation are reported in Table 2, while the gains reported in [7] are used for SMC and STW computer simulation as well as in practical implementation. The simulation is performed for the reference tracking of fixed point *r*_*d*_(*t*). The desired position is selected to be 22*cm* on a beam. (representing in Eq (39)). For the simulation, initial condition is set to be *x*_1_(0) = 0.4, the rest of the state variables are considered zero initially.

$$r_{d}(t)=22cm\;t>0$$ (39)

**Fig 2:**
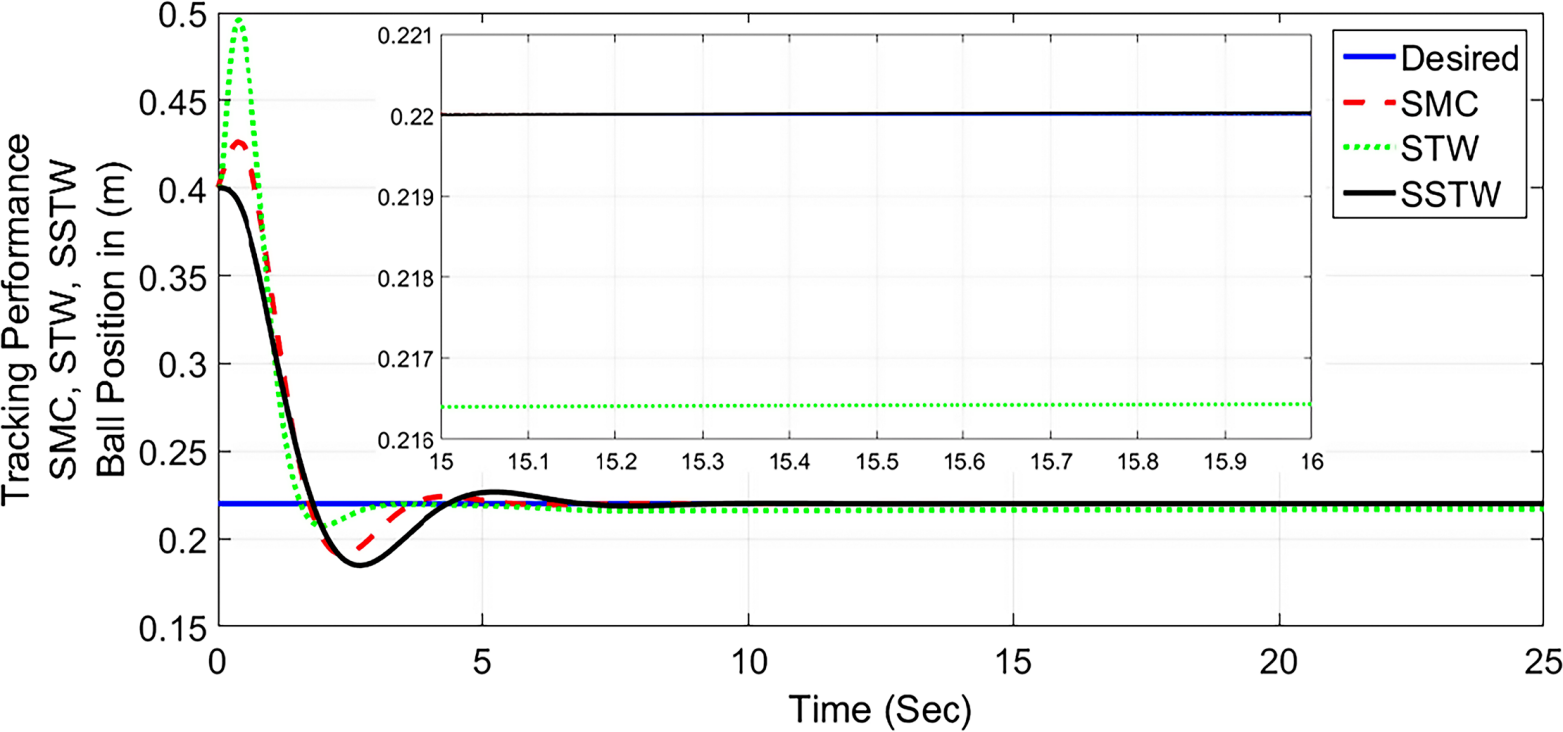
Output tracking performance of SMC, STW, and SSTW, *r*_*d*_ = 22*cm*.

**Fig 3:**
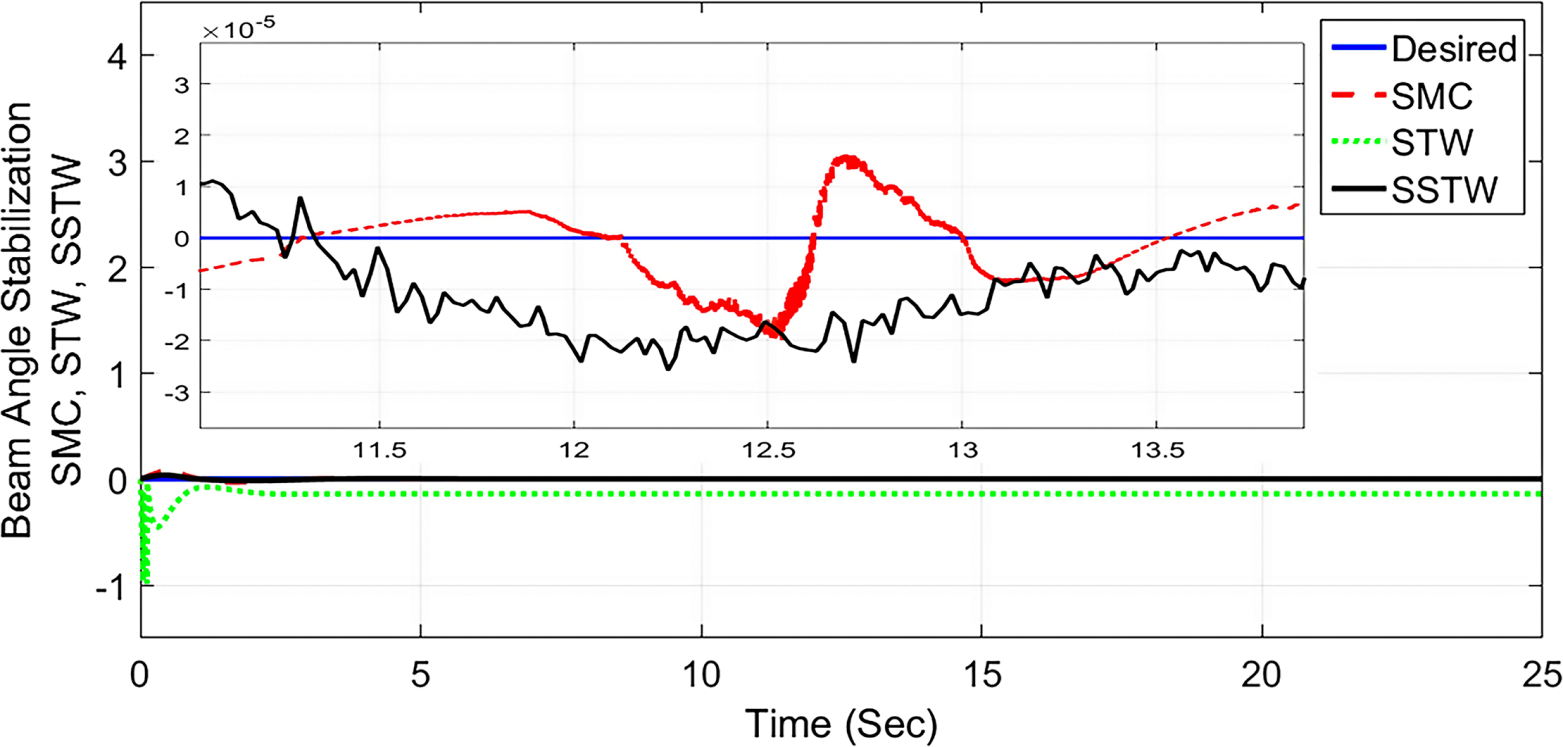
Beam angle stabilization profile of SMC, STW and SSTW.

**Fig 4:**
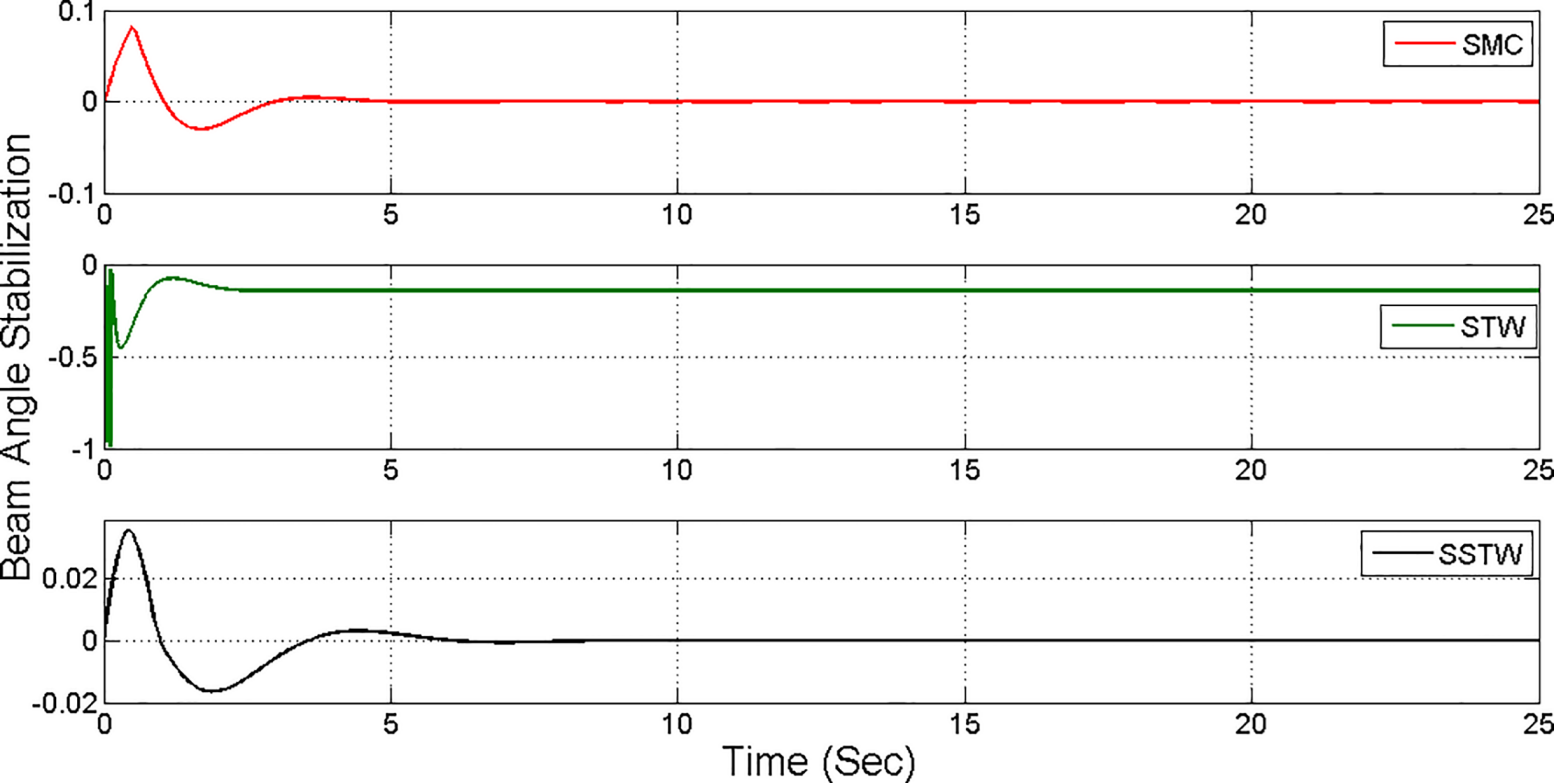
Separate profile regarding stabilization of beam angle SMC, STW and SSTW.

The output tracking performance of SSTWSMC along with STW and SMC is shown in (Fig 2). It can be clearly examined that the tracking performance of SSTW is very precise with no overshoot as compared to SMC and STW. The magnified view of reference tracking highlights that the precision of the STW is not very appealing as compared to SMC and SSTWSMC. The beam angle stabilization profile for SMC, STW and SSTWSMC are displayed in (Fig 3) along with their magnified version. The separated profile of the beam angle stabilization can be seen in (Fig 4). The magnified version of the angle stabilization shows the steady state error in case of STW as compared to the other stabilization strategies. SMC and SSTWSMC show impressive results in view point of beam angle stabilization.

**Fig 5:**
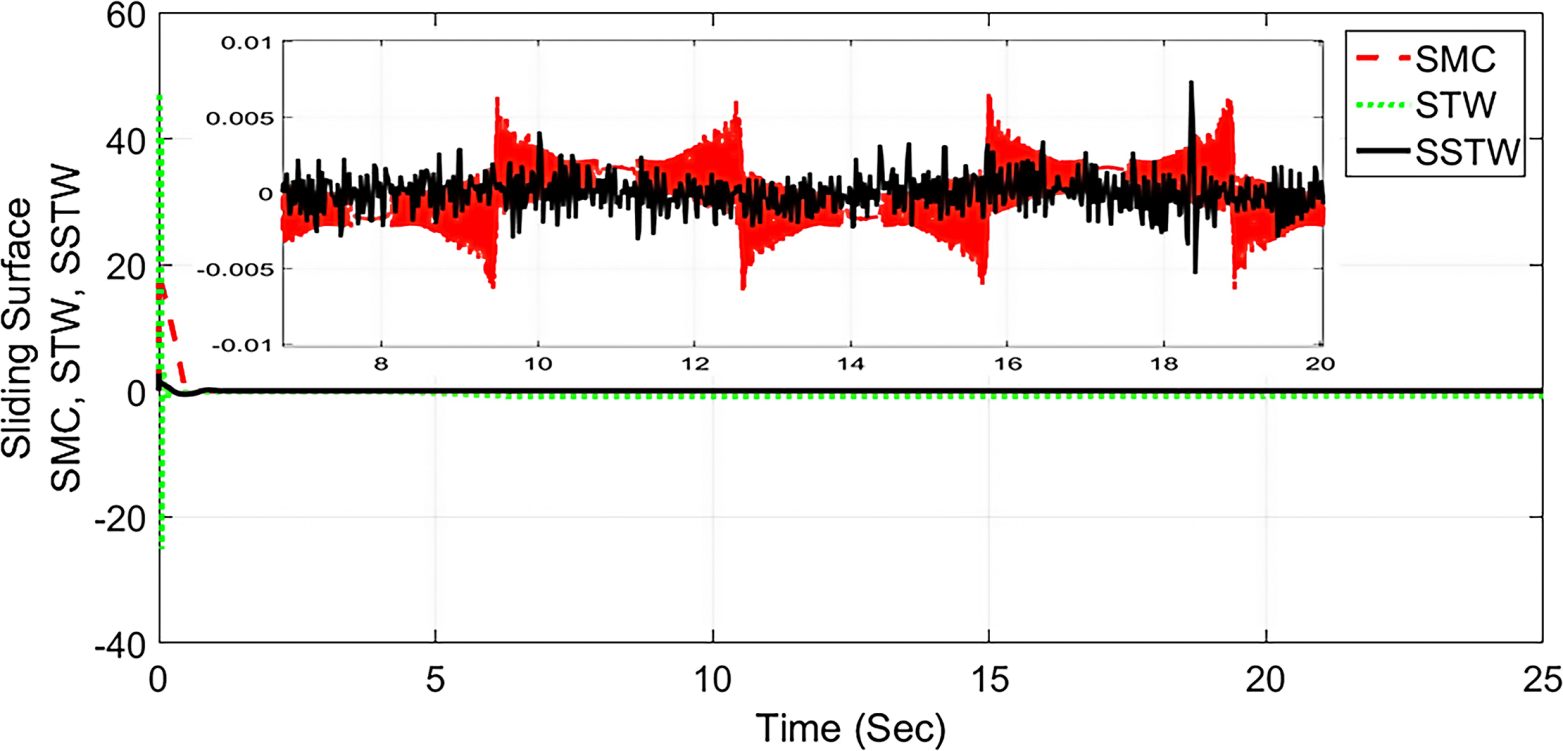
Sliding manifold convergence profile of SMC, STW and SSTW, *r*_*d*_ = 22*cm*.

**Fig 6:**
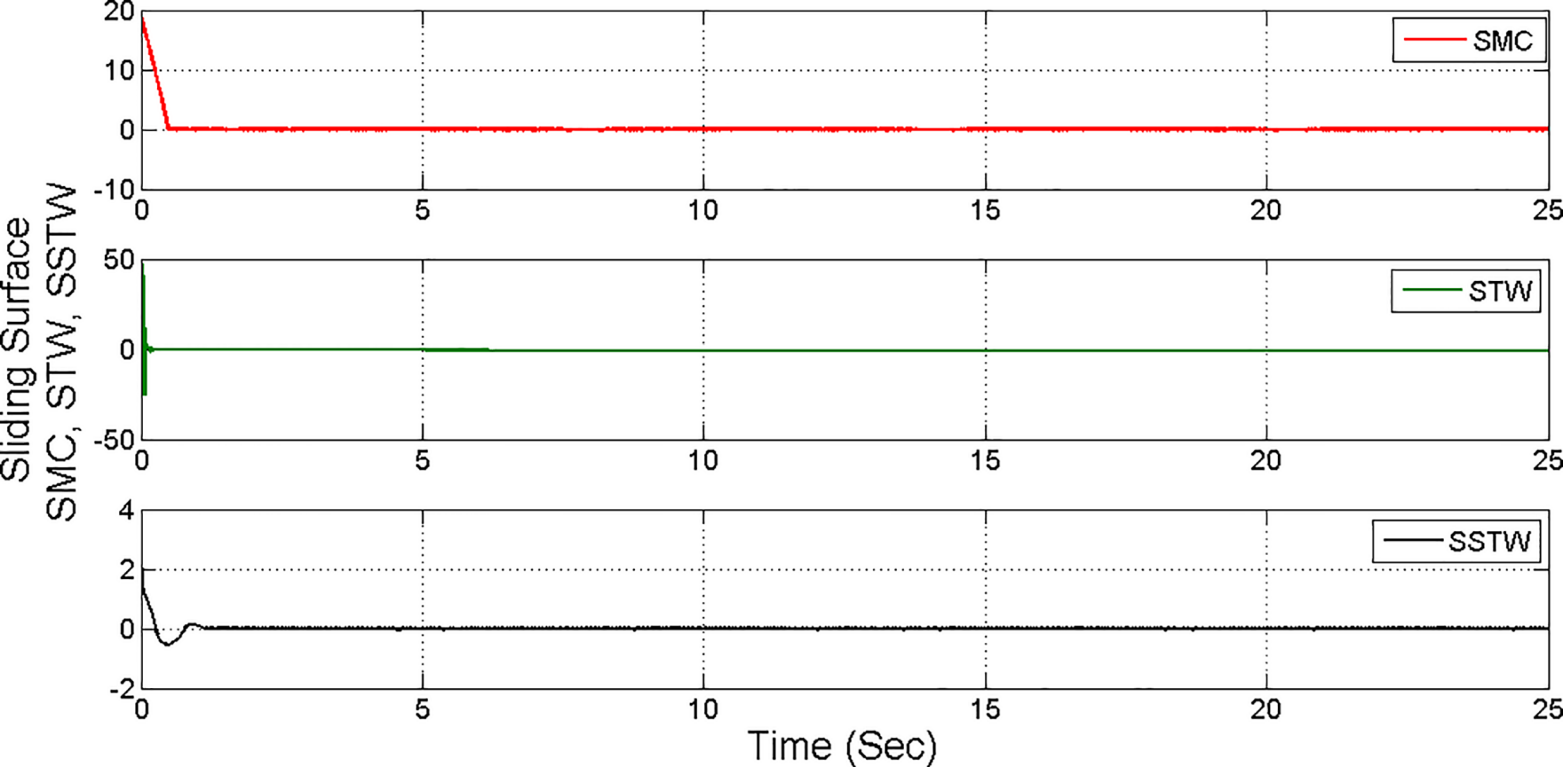
Separate sliding manifold convergence profile of SMC, STW and SSTW.

**Fig 7:**
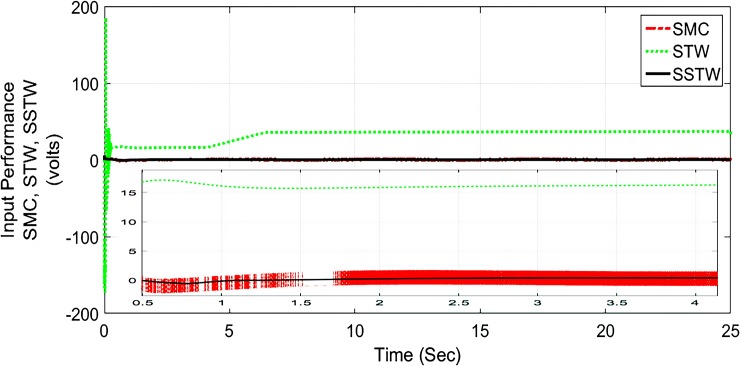
Control Input profile of SMC, STW and SSTW for reference tracking.

**Fig 8:**
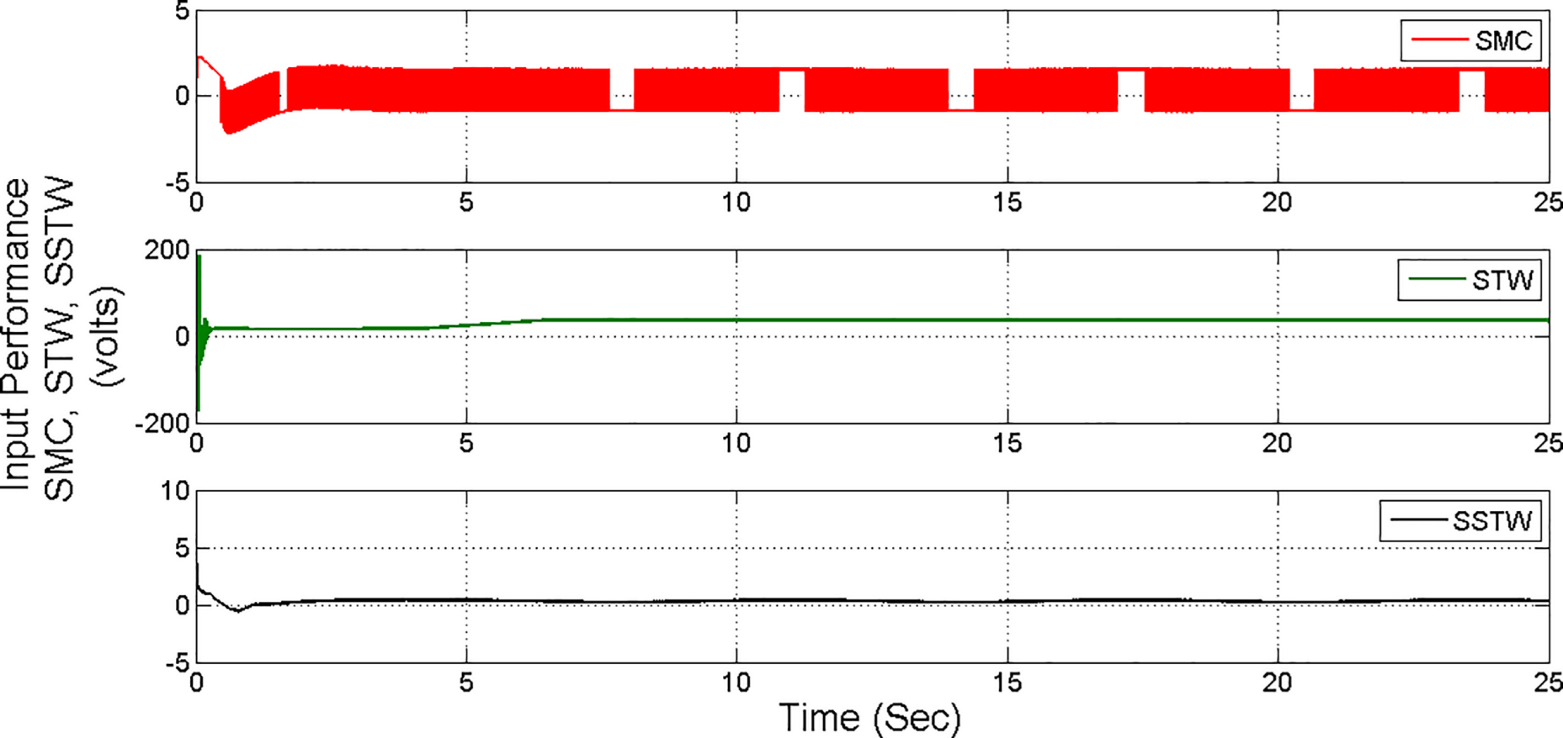
Separate control input profile of SMC, STW and SSTW.

However, SMC suffers from severe chattering phenomenon, which can be seen clearly in (Fig 5) magnified profile. As compared to SMC, SSTWSMC appears to be very appealing (due to low oscillatory behavior). Figs 5 and 6 display the sliding manifold profiles, their respective control input is shown in Figs 7 and 8 for the aforesaid three strategies respectively.

**Fig 9:**
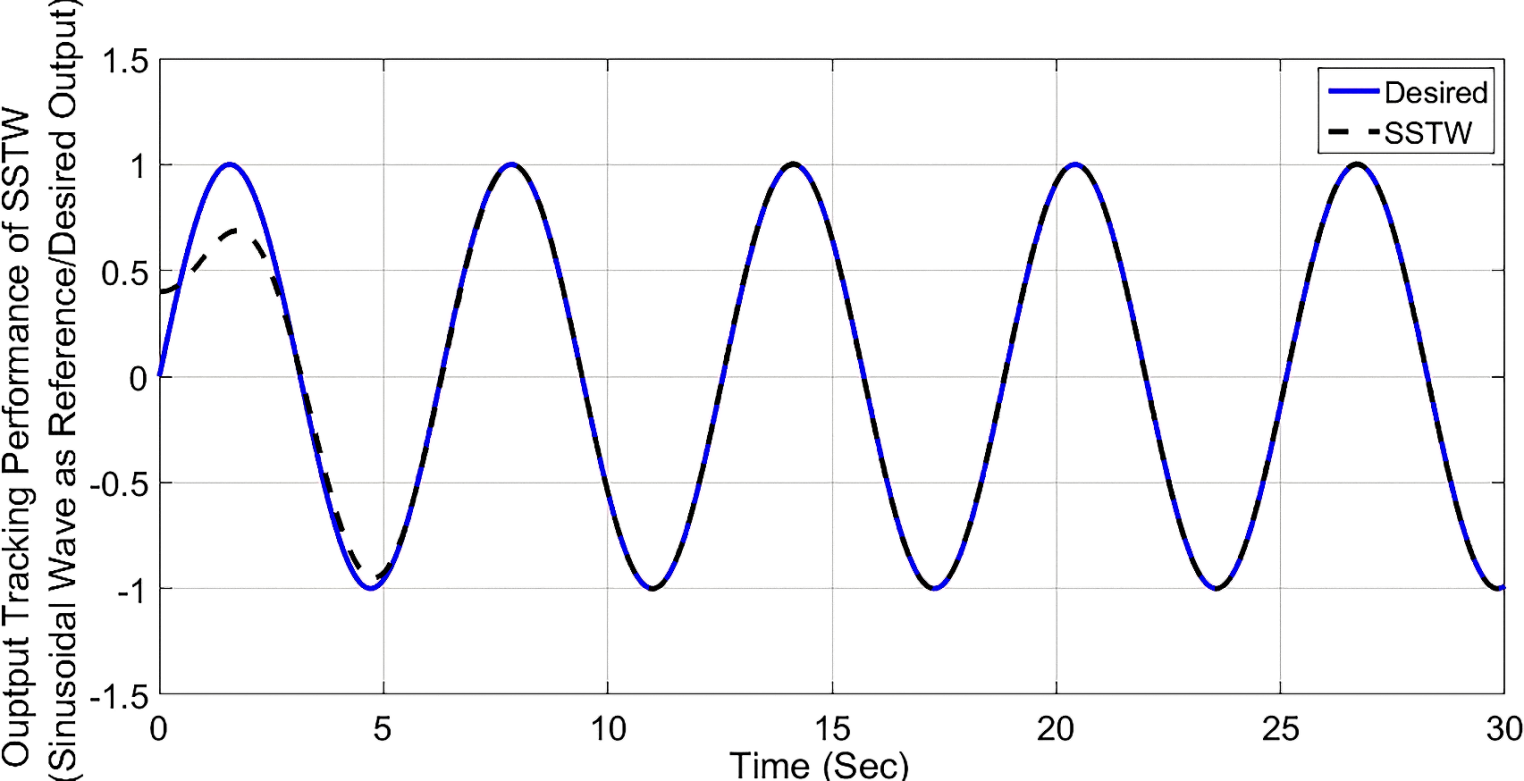
Output tracking performance of SSTW, considering sinusoidal reference input signal.

**Fig 10:**
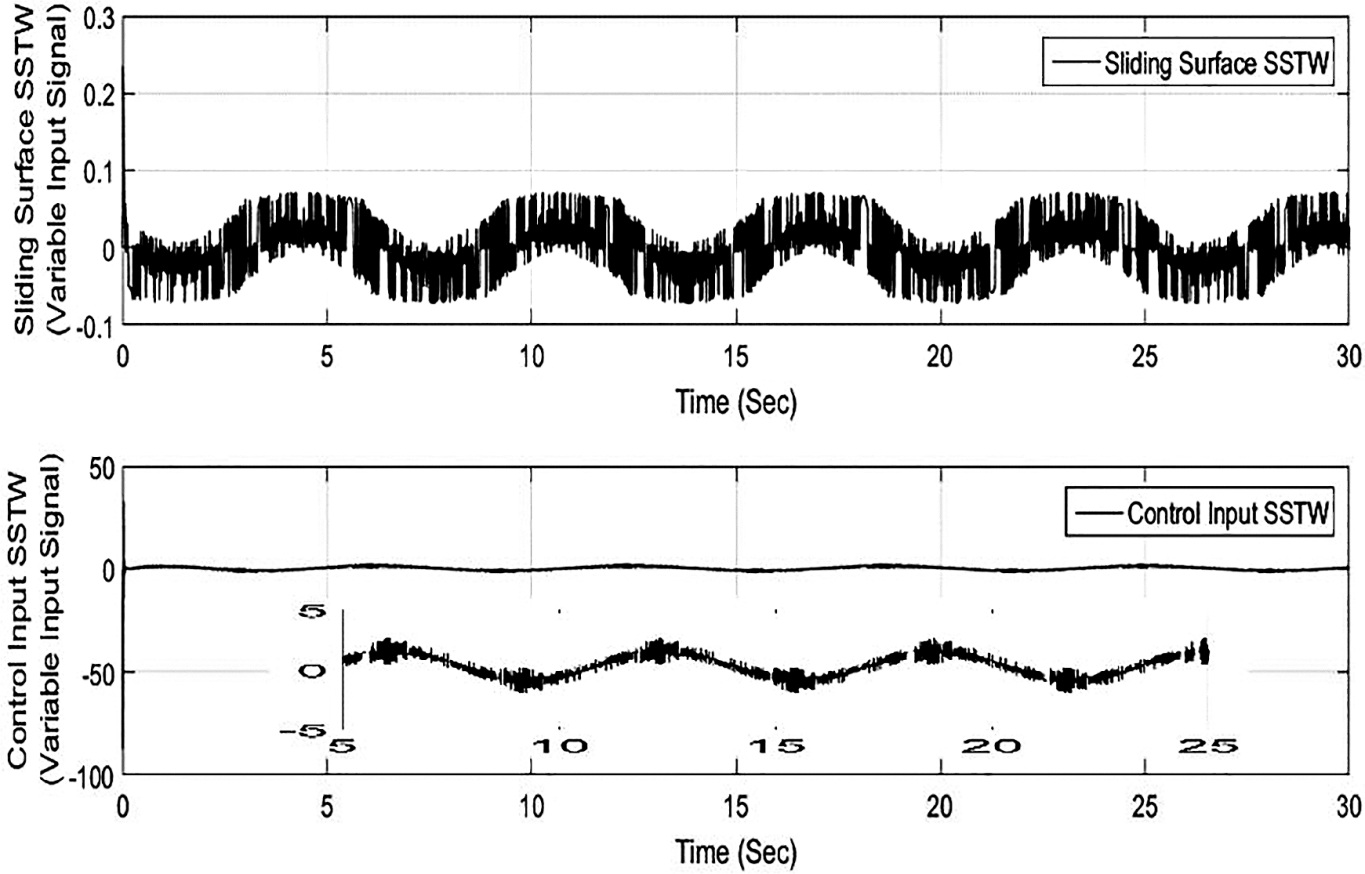
Sliding Surface and Control Input profile for SSTW, considering sinusoidal reference input signal.

Considering from Figs 2–8, simulation analysis indicates the SSTW outshines the other techniques in more attributes. Therefore, SSTW is also tested for the sinusoidal input (variable reference signal). (Fig 9), shows the tracking profile of the SSTW with sinusoidal reference input. It can be clearly seen that the performance is excellent in this scenario. Corresponding sliding surface and control effort is also displayed in (Fig 10), significantly confirms the establishment of sliding mode. It is obvious from the figure that the control input evolves with suppressed chattering phenomenon which, once again, makes this design strategy a good candidate for the class of these underactuated systems.

#### Remark 3

From (3), ΔG_m_(ξ,t) = 0.5 Sin(t) is considered as a matched disturbance. This effect is considered during the simulation results shown in Figs 2–10. In experimentation setup Googoltech (GBB1004), the effect of noise / matched disturbances are already included. The (S1 File. Data files) contains the simulation and experimental data regarding the attributes (tracking performance, control input, sliding surface and chattering analysis/beam angle stabilization mentioned in Table 3.

**Table 3:** Comparative analysis SMC, STW and SSTWSMC.

Attributes	FOSMC	STW	SSTWSMC
**Tracking Control**	Moderate (Not precise)	Fast (Not precise)	Fast (Precise)
**Settling Time**	Normal	Low	Normal
**Overshoot**	High	Very high (Maximum)	No Overshoot
**Chattering Analysis**	Severe chattering	Low chattering	Minimal chattering
**Sliding Surface Convergence**	To origin, with the chattering of medium magnitude amplitude	Remains at the origin with small magnitude oscillations in the very start	To origin, with moderate chattering amplitude
**Control Effort**	Very high	High	Minimum
**Computational** **Complexity**	Low	High	High

### Experimental results

**Fig 11:**
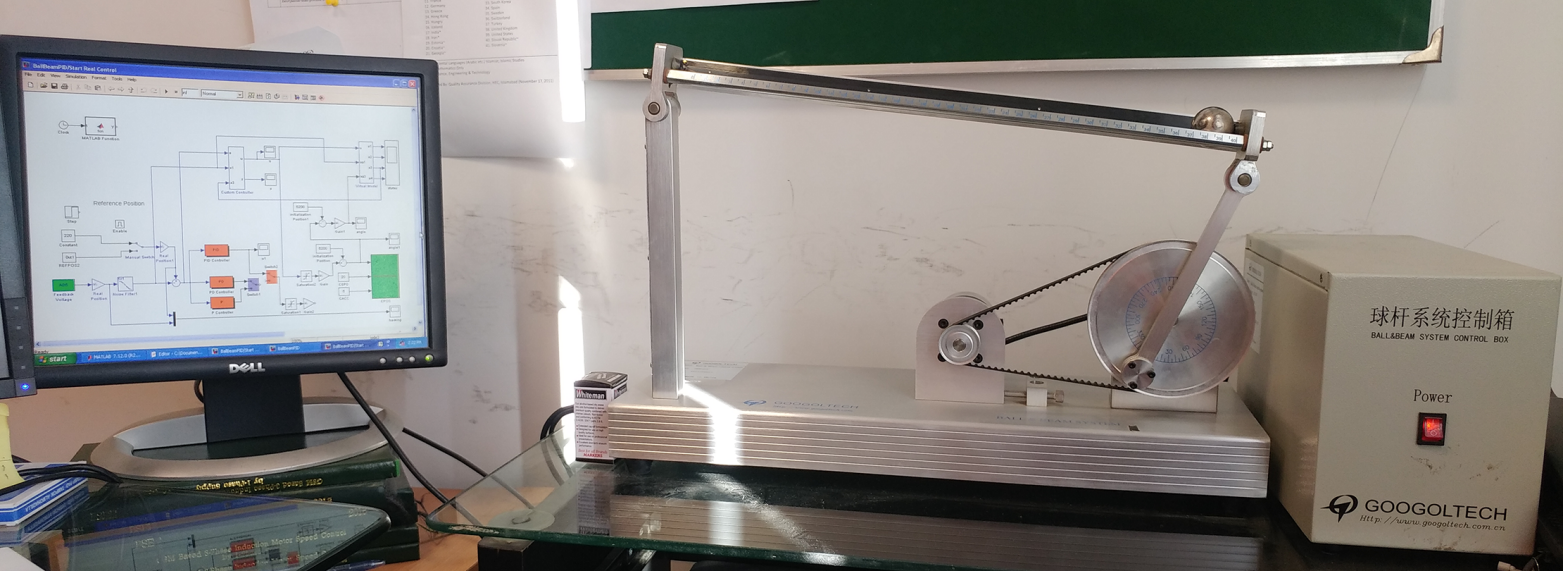
Googoltech GBB1004, Ball and beam balancer experimental framework.

As for the implementation, the core objective is to track the position of the ball at the desired position *r*_*d*_ (as mentioned in (39)). The Control scheme proposed in Section III is applied to the ball and beam balancer. This equipment is manufactured by Googoltech Technology (GBB1004), displays in (Fig 11). It consists of IPM100 intelligent servo drive electronic control box, which requires an operating current of 10A with the voltage of 220V. The beauty of the aforesaid control box is its interfacing to MATLAB 7.12 and Simulink 7.7 framework. The beam (metallic) of the experimental system is 40cm in length carries the ball (metallic) of weight approximately 40g. The servo motor used in this system is quite capable to rotate the beam, clockwise/anticlockwise (as required) It is worthy to mention here, that the control accuracy or precision of this equipment falls with in ±1*mm*. The Sampling time of 2*ms* is considered during experimentation. In order to make the experimentation easy and convenient, Simulink built-in time derivative block is used for corresponding velocity measurements.

**Fig 12:**
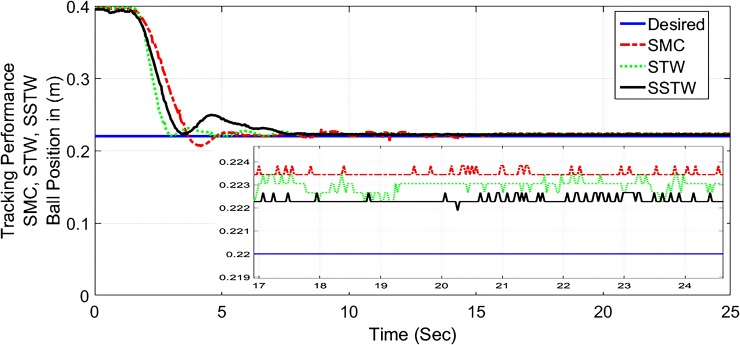
Output tracking performance of SMC, STW, and SSTW, *r*_*d*_ = 22*cm*.

**Fig 13:**
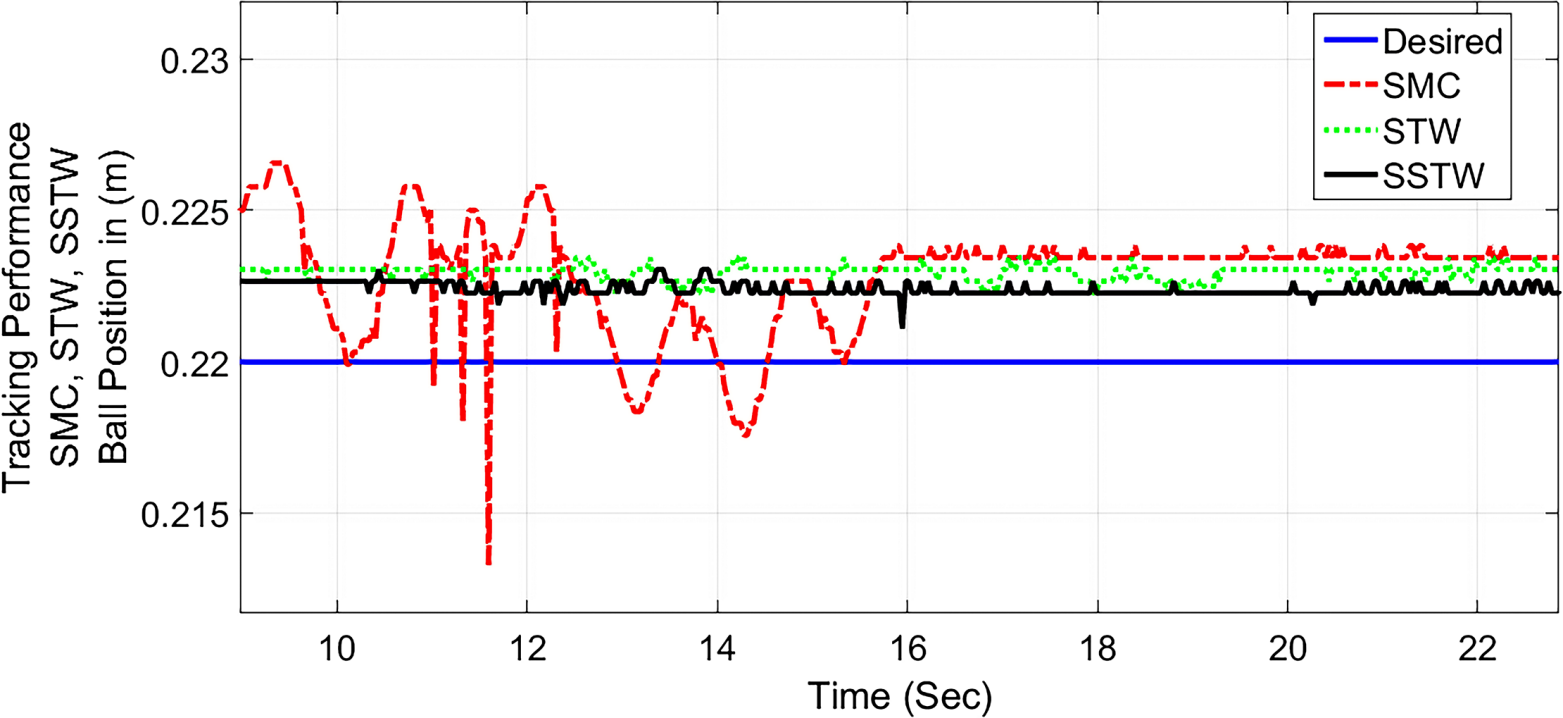
Chattering suppression and output tracking performance of SMC, STW and SSTW, *r*_*d*_ = 22*cm*.

In this implementation, *r*_*d*_ is selected to be 22*cm* mark present on the metallic beam. As simulation result of SSTWSMC is extensively analyzed with the simulation results of SMC and SSTW laid in [7], similarly, following the same footprint, the experimental study of SSTWSMC is also comparing the experimental results of conventional SMC and STW posed in [7]. (Fig 12) portrays the tracking profile of SMC, STW and SSTWSMC, where (Fig 13) displays its magnified version.

**Fig 14:**
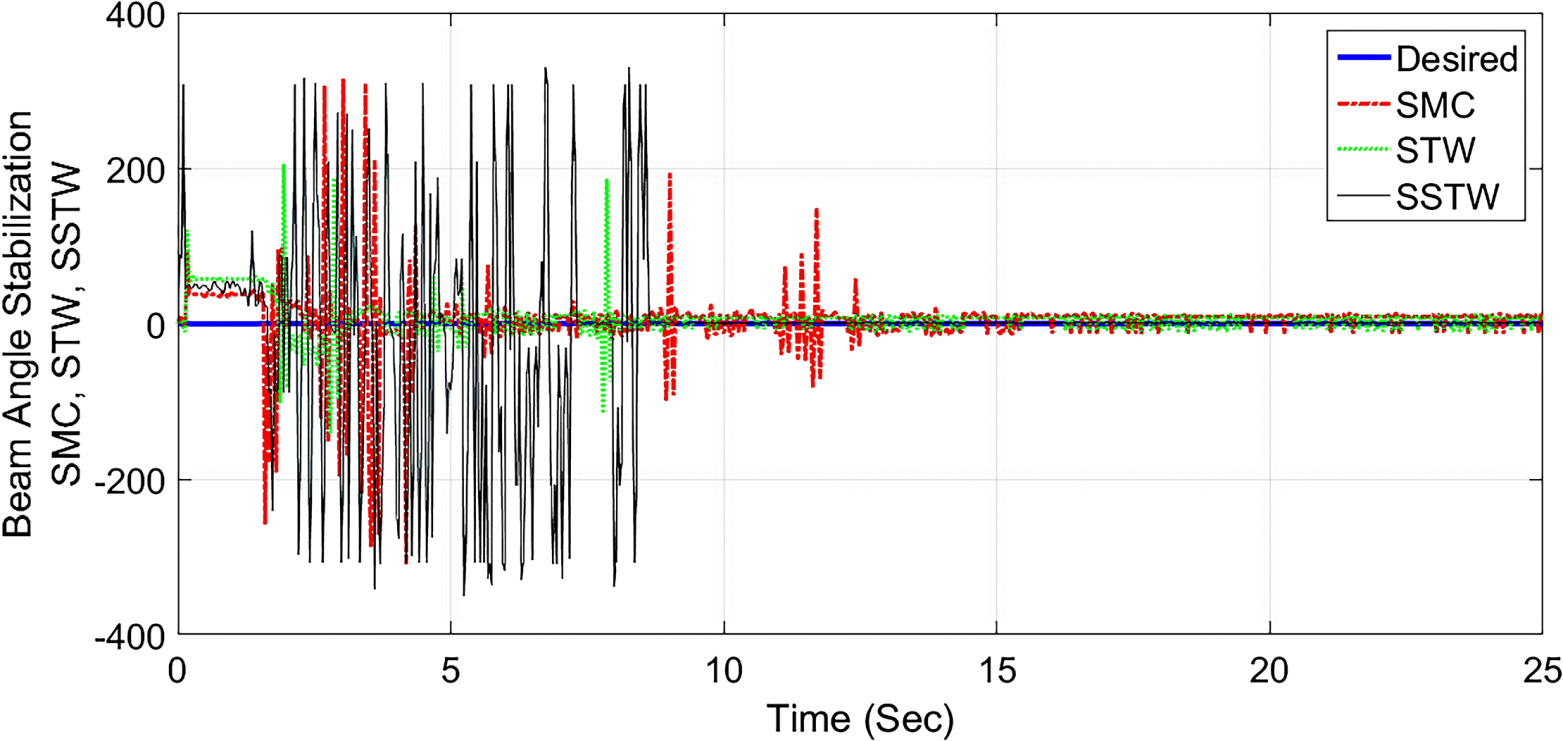
Beam angle stabilization profile of SMC, STW and SSTW.

**Fig 15:**
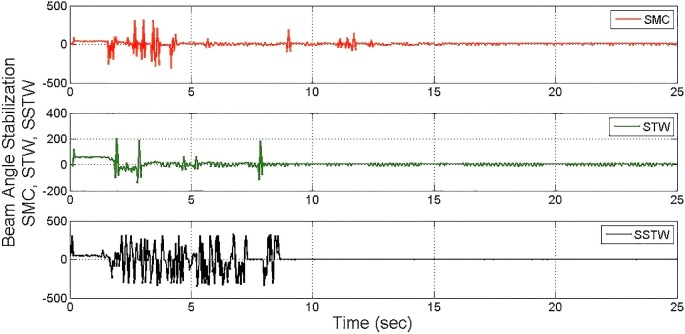
Individual profile regarding stabilization of beam angle SMC, STW and SSTW.

It is evident that the SSTWSMC shows the most precise results toward the desired point and minor deviation exists due to hardware limitation. STW stands at mark two and conventional SMC stands third with respect to precession/accuracy. Chattering is considerably high in SMC, moderate in STW, minimal in SSTWSMC (can be examined by (Fig 13). Stabilization profile of beam is presented in (Fig 14), which displays chattering in SSTWSMC as compared to SMC and STW. It is also worthy to note that, when *r*_*d*_ is achieved, SSTWSMC is smoother than other two techniques (it can be observed from their individual profile in (Fig 15)).

**Fig 16:**
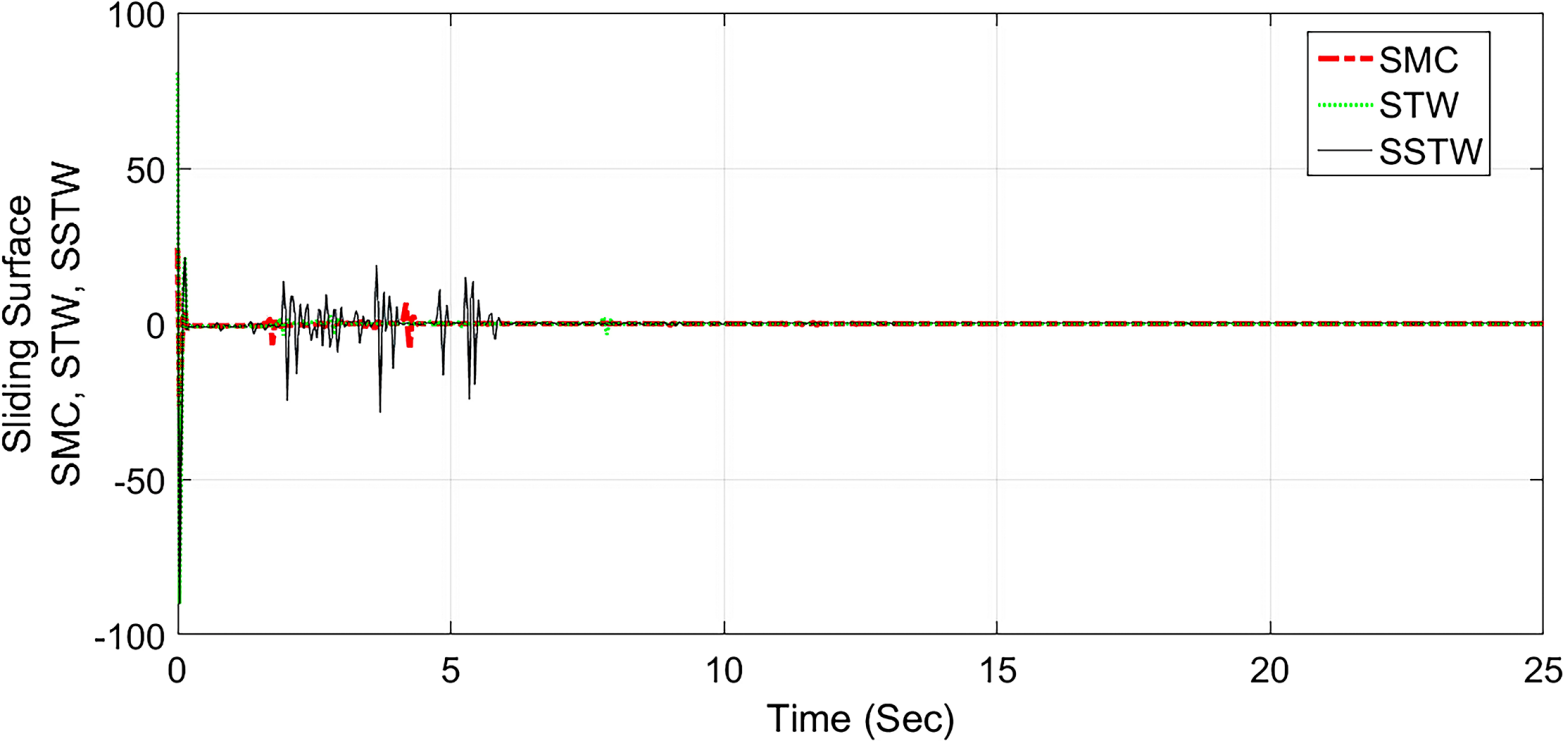
Sliding manifold convergence profile of SMC, STW and SSTW, *r*_*d*_ = 22*cm*.

Sliding manifold convergence of SMC, STW, and SSTWSMC collectively displayed in (Fig 16), it can be observed they have stable manifold converging towards the origin.

**Fig 17:**
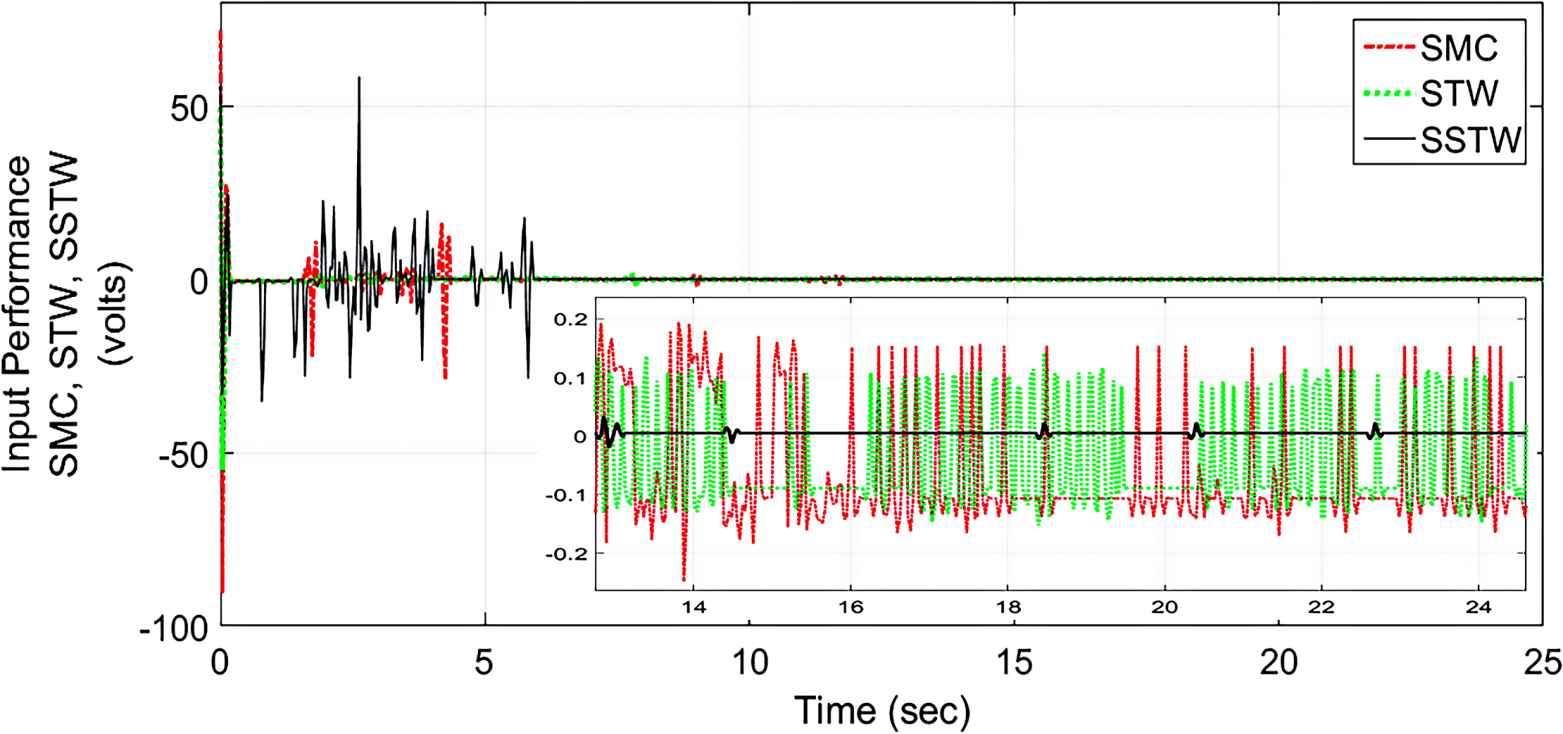
Control Input profile of SMC, STW and SSTW for reference tracking.

**Fig 18:**
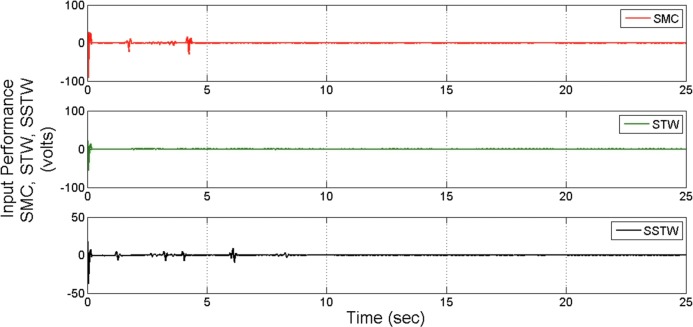
Individual profile regarding control input of SMC, STW and SSTW.

Figs 17 and 18 display the control input performance of the ball and beam balancer regarding tracking of *r*_*d*_ From (Fig 17), it can be concluded that SSTW based SMC is utilizing minimum energy as compare to the other two strategies. Collectively, considering all the attributes, we can conclude that the application of SMC in real systems is limited due to high energy utilization and higher amplitude of oscillations. STW has low chattering, but high control effort is required to keep it that way. Continuously control effort is required even after the desired position is achieved (can be seen in (Fig 17)), on the other hand, SSTW based SMC has higher precision among SMC and STW. Chattering is almost negligible in SSTWSMC, after the desired position is achieved (during pursuing *r*_*d*_, it is assumed that it should remain in the allowable tolerance level). In addition to this, it utilizes minimum control effort (can be observed from (Fig 17)). Table 3 summarizes the extensive comparative analysis.

In accordance with the attributes presented in Table 3, it can be concluded, that SSTW based SMC outshines the other two techniques in sufficient parameters.

## Conclusion

The proposed technique of Smooth Super-Twisting Sliding Mode Control Sliding Mode Control (SSTWSMC), performs up to the mark for the class of underactuated system. Before design procedure, the nonlinear system is transformed into a controllable canonical form; then the controller is designed via SSTWSMC. MATLAB/SIMULINK environment is being used to conduct the simulation and experimental work. For critical analysis, SSTWSMC is comparatively studied with the STW and SMC. After analysis, it is concluded that SSTWSMC have suppressed chattering and it can deliver robust tracking performance with minimal energy utilization. SSTWSMC outshines the SMC and STW in a maximum number of attributes. The benefit of this study is to analyse the appealing attitude of SSTWSMC in the electromechanical systems.

From the perspective of future research regarding the class of underactuated systems, Adaptive Global Sliding Mode Control (AGSMC) [12] and LMI based GSMC [14] should be considered.

## Supporting information

S1 File Data files. (RAR)
